# Automatic morphology phenotyping of tetra- and hexaploid wheat spike using computer vision methods

**DOI:** 10.18699/VJ21.009

**Published:** 2021-02

**Authors:** A.Yu. Pronozin, A.A. Paulish, E.A. Zavarzin, A.Yu. Prikhodko, N.M. Prokhoshin, Yu.V. Kruchinina, N.P. Goncharov, E.G. Komyshev, M.A. Genaev

**Affiliations:** Institute of Cytology and Genetics of Siberian Branch of the Russian Academy of Sciences, Novosibirsk, Russia; Novosibirsk State University, Novosibirsk, Russia; Novosibirsk State University, Novosibirsk, Russia; Novosibirsk State University, Novosibirsk, Russia; Novosibirsk State University, Novosibirsk, Russia; Institute of Cytology and Genetics of Siberian Branch of the Russian Academy of Sciences, Novosibirsk, Russia Kurchatov Genomics Center of the Institute of Cytology and Genetics of Siberian Branch of the Russian Academy of Sciences, Novosibirsk, Russia; Institute of Cytology and Genetics of Siberian Branch of the Russian Academy of Sciences, Novosibirsk, Russia Novosibirsk State Agrarian University, Novosibirsk, Russia; Institute of Cytology and Genetics of Siberian Branch of the Russian Academy of Sciences, Novosibirsk, Russia Novosibirsk State University, Novosibirsk, Russia Kurchatov Genomics Center of the Institute of Cytology and Genetics of Siberian Branch of the Russian Academy of Sciences, Novosibirsk, Russia; Institute of Cytology and Genetics of Siberian Branch of the Russian Academy of Sciences, Novosibirsk, Russia Novosibirsk State University, Novosibirsk, Russia Kurchatov Genomics Center of the Institute of Cytology and Genetics of Siberian Branch of the Russian Academy of Sciences, Novosibirsk, Russia

**Keywords:** wheat spike morphology, wheat, phenomics, image processing, computer vision, machine learning, biotechnology, пшеница, морфология колоса, феномика, обработка изображений, компьютерное зрение, машинное обучение, биотехнологии

## Abstract

Intraspecific classification of cultivated plants is necessary for the conservation of biological diversity, study of their origin and their phylogeny. The modern cultivated wheat species originated from three wild diploid ancestors as a result of several rounds of genome doubling and are represented by di-, tetra- and hexaploid species. The identification of wheat ploidy level is one of the main stages of their taxonomy. Such classification is possible based on visual analysis of the wheat spike traits. The aim of this study is to investigate the morphological characteristics of spikes for hexa- and tetraploid wheat species based on the method of high-performance phenotyping. Phenotyping of the quantitative characteristics of the spike of 17 wheat species (595 plants, 3348 images), including eight tetraploids (Triticum aethiopicum, T. dicoccoides, T. dicoccum, T. durum, T. militinae, T. polonicum, T. timopheevii, and T. turgidum) and nine hexaploids (T. compactum, T. aestivum, i:ANK-23 (near-isogenic line of T. aestivum cv. Novosibirskaya 67), T. antiquorum, T. spelta (including cv. Rother Sommer Kolben), T. petropavlovskyi, T. yunnanense, T. macha, T. sphaerococcum, and T. vavilovii), was performed. Wheat spike morphology was described on the basis of nine quantitative traits including shape, size and awns area of the spike. The traits were obtained as a result of image analysis using the WERecognizer program. A cluster analysis of plants according to the characteristics of the spike shape and comparison of their distributions in tetraploid and hexaploid species showed a higher variability of traits in hexaploid species compared to tetraploid ones. At the same time, the species themselves form two clusters in the visual characteristics of the spike. One type is predominantly hexaploid species (with the exception of one tetraploid, T. dicoccoides). The other group includes tetraploid ones (with the exception of three hexaploid ones, T. compactum, T. antiquorum, T. sphaerococcum, and i:ANK-23). Thus, it has been shown that the morphological characteristics of spikes for hexaploid and tetraploid wheat species, obtained on the basis of computer analysis of images, include differences, which are further used to develop methods for plant classifications by ploidy level and their species in an automatic mode.

## Abstract (ru)

Внутривидовая классификация культурных растений необходима для эффективного сохранения биологического разнообразия видов, изучения их происхождения, определения филогении и проведения межвидовой гибридизации при селекции. Современные возделываемые виды пшениц произошли от трех диких диплоидных предков в результате гибридизации и нескольких раундов удвоения геномов и представлены ди-, тетра- и гексаплоидными видами. Поэтому идентификация плоидности пшениц и определение их геномного состава являются одними из основных этапов их классификации на основе визуального анализа фенотипических признаков колоса. Цель работы – исследование морфологических характеристик колосьев полиплоидных видов пшеницы методами высокопроизводительного фенотипирования. Выполнено фенотипирование количественных характеристик колоса 17 видов пшеницы (595 растений, 3348 изображений), включая восемь тетраплоидных: Triticum aethiopicum, T. dicoccoides, T. dicoccum, T. durum, T. militinae, T. polonicum, T. timopheevii, T. turgidum и девять гексаплоидных: T. compactum, T. aestivum (в том числе изогенная линия сорта Новосибирская 67 АНК-23), T. antiquorum, T. spelta (включая стародавний сорт T. spelta Rother Sommer Kolben), T. petropavlovskyi, T. yunnanense, T. macha, T. sphaerococcum, T. vavilovii. Морфология колоса описана на основе девяти количественных признаков, включающих форму, размер и остистость. Признаки были получены в результате анализа цифровых изображений с помощью программы WERecognizer. Кластерный анализ растений по характеристикам формы колоса и сравнение их распределений у тетра- и гексаплоидных видов показали более высокую вариабельность признаков у гексаплоидных видов по сравнению с тетраплоидными. При этом сами виды в пространстве характеристик колоса формируют два кластера. К первому относятся преимущественно гексаплоидные виды, за исключением одного тетраплоидного, дикорастущего T. dicoccoides, ко второму – тетраплоидные, за исключением трех гексаплоидных, T. compactum, T. antiquorum, T. sphaerococcum, и i:АНК-23. Показано, что морфологические характеристики колосьев для гекса- и тетраплоидных видов, полученные на основе компьютерного анализа изображений, демонстрируют различия, которые в дальнейшем могут быть использованы для разработки методики эффективной классификации растений по плоидности и их видовой принадлежности в автоматическом режиме.

## Introduction

A number of important issues, including aspects of the effective conservation of the biological diversity of cultivated plant species, the study of their origin, and their phylogeny, presupposes a detailed development of intraspecific classifications (Dorofeev et al., 1979; Goncharov, 2011). The producing of such classifications, reflecting the phylogenesis and genetic structure of species, should be considered the main goal of modern taxonomy (Hammer et al., 2011). When developing the classification of cultivated plants, the most complete description of all existing large and small forms (taxons) is assumed (Sinskaya, 1969). On the one hand, this is determined, by the convenience of using such a division in experimental work, on the other hand, it is also determened in the breeding and testing of cultivated plants. 

The success and effectiveness of research work is often associated with the detailing and completeness of the experimental study, which depends on what the material is and how much it should be studied. In this regard, it is extremely important that the natural differentiation of one or another genus, the relationship between species, are reflected with high accuracy by a detailed taxonomy (Dorofeev, 1985). It should be noted, that for most of the plants important for agriculture, the volumes of the genus and species have not been unambiguously described yet (Rodionov et al., 2019).

A serious problem in the taxonomy of cultivated plants is the aspect of taxa agregation vs. fragmentation, and in cases of cultivation it manifests itself especially in contrast (Golovnina et al., 2009; Goncharov, 2011). At the same time, the effective use of taxonomy of cultivated plants in the work of researchers causes certain difficulties. Both dichotomous tables (Dorofeev et al., 1979; Goncharov, 2009) and ideographic manual book (Zuev et al., 2019) require certain skills; therefore, the producing of a database and software that allows the identification of species by digital images is a very promising direction. The development of these methods is mainly based on technologies for analyzing digital images of plant organs within the framework of computer phenomics (Afonnikov et al., 2016; Zhang et al., 2019; Demidchik et al., 2020; Yang et al., 2020).

Wheat is one of the world’s most important food crops. The modern cultivated wheat species evolved from three wild diploid ancestors as a result of their hybridization and several rounds of genome doubling (polyploidization). Currently, cultivated wheat is represented by di- (2n = 2x = 14, AbAb genome), tetra- (2n = 4x = 28, BBAuAuDD genome) and hexaploid (2n = 6x = 42, BBAuAuDD genome) species (Goncharov, Kondratenko, 2008). The main cultivated species, bread wheat (Triticum aestivum L.), is a hexaploid (genomic formula BBAuAuDD). The ploidy level is one of the main taxonomic and classifying characteristics of wheat species (Dorofeev et al., 1984; van Slageren, Payne, 2013). It can be established by cytogenetic (Rodionov et al., 2020), molecular methods, as well as by comparing the morphological characteristics of plants (Dorofeev et al., 1984). In this work, we studied the morphological traits of the plant spikes of tetraploid and hexaploid wheat species based on the method of high-throughput phenotyping.

The aim of the research was to study the distribution of morphological traits of spikes of tetra- and hexaploid wheat species and compare their distributions.

## Material and methods

**Biological material.** We studied 17 polyploid wheat species, namely, nine hexaploids (Triticum compactum Host, T. aestivum L., T. antiquorum Heer ex Udacz., T. spelta L. (includion of T. spelta cv. Rother Sommer Kolben), T. petropavlovskyi Udacz. et Migusch., T. yunnanense King ex S.L. Chen, T. macha Dekapr. et Menabde, T. sphaerococcum Perciv., T. vavilovii (Thum.) Jakubz.), the near-isogenic line ANK-23 of bread wheat cv. Novosibirskaya 67 and eight tetraploids (T. aethiopicum Jakubz., T. dicoccoides (Körn. ex Aschers. et Graebn.) Schweinf., T. dicoccum (Schrank) Schuebl., T. durum Desf., T. militinae Zhuk. et Migusch., T. polonicum L., T. timopheevii (Zhuk.) Zhuk., T. turgidum L.); the sample consists of spikes of 595 individual plants, which was grown in nine vegetation seasons. The plants were grown in 2014–2019 in a greenhouse at the Shared Center ‘Laboratory of Artificial Plant Cultivation’ of the Institute of Cytology and Genetics SB RAS. A description of the material used is given in Table 1.

**Table 1.**
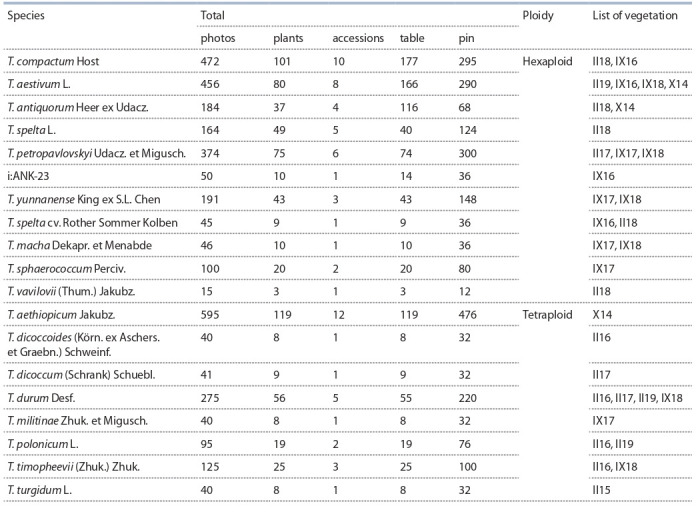
Characteristics of the studied wheat species

It should be noted that none of the large genebanks of the world have typical sets of wheat accessions (collections), so them usually reflect either the researchers view on the methods of selection of such sets (Palmova, 1935) or are determined by the representativeness of the researchers available material (Goncharov, Shumny, 2008). Standard taxonomic descriptions of specimens are given in publicly available databases on genebank websites (http://db.vir.nw.ru/virdb/maindb)

**Fig. 1.**
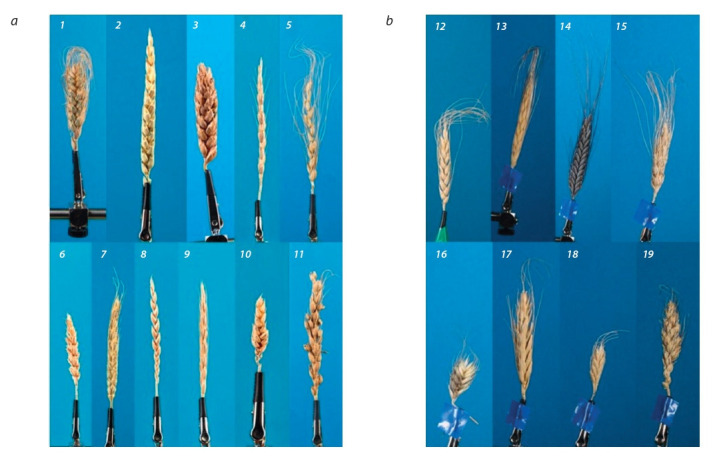
Spike images of hexaploid (a) and tetraploid (b) wheat species. 1 – T. compactum; 2 – T. aestivum; 3 – T. antiquorum; 4 – T. spelta; 5 – T. petropavlovskyi; 6 – i:АNК-23; 7 – T. yunnanense; 8 – T. spelta cv. Rother Sommer Kolben; 9 – T. macha; 10 – T. sphaerococcum; 11 – T. vavilovii; 12 – T. aethiopicum; 13 – T. dicoccoides; 14 – T. dicoccum; 15 – T. durum; 16 – T. militinae; 17 – T. polonicum; 18 – T. timopheevii; 19 – T. turgidum.

**Digital images obtaining.** In this work we used two protocols for receiving mature spikes photo. The first is that the spike is placed on the glass of a light table, which is located on a table with a blue top (background). The camera is fixed on a stand above the glass. With this method, the front projection of the spike can be captured. Second, the spike is held vertically in front of the blue background. The spike is supported by clip that are placed on a tripod. With this method, by rotating the spike about its axis, four or more projections of the spike can be captured (Genaev et al., 2018). According to the protocols, a ColorChecker must be present in the photographs. It is needed for colour normalisation and scaling. One plant in our dataset can correspond to up to five pictures of its spike taken with different protocols and in different projections. Examples of spikes images (one for each species) are shown in Fig. 1. In total 3348 spike images in different projections were captured by the two protocols, 2097 of them were of hexaploid species and 1251 were of tetraploid species. Of these, 915 images were obtained using the “on the table” protocol and 2433 “on the clip”.

**Fig. 2.**
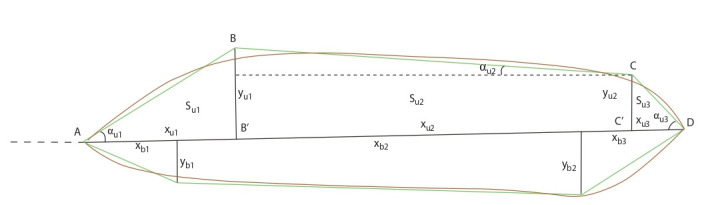
Wheat spike shape represents in the form of two quadrangles (Genaev et al., 2019) The black horizontal line shows the spike centerline. Brown line – spike contour. Green lines – the quadrilaterals that approximate the spike contour. The spike base – left dotted line. The figure for the upper quadrangle shows the main parameters that characterize spike geometry. Similar parameters are defined for the lower quadrangle.

Evaluation of spikes quantitative characteristics. WERecognizer (Genaev et al., 2019) was used to estimate spikes quantitative characteristics based on image analysis. This program describes a wheat spike by geometric model of two quadrangles based on image analysis (Fig. 2). The geometry of this model is described by nine independent parameters. The parameters xu1, xu2, yu1, yu2 are for the upper quadrangle; the parameters xb1, xb2, yb1, yb2 are for the lower quadrangle; the common parameter for the two quadrangles is the ear length. The program additionally calculates a number of general features of the shape and size of the spike, as well as the characteristic of its awning. Details of the feature extraction algorithm are given in (Genaev et al., 2019).

In the present study, we used the model traits that we selected as the most informative for predicting spike density index in our previous study (Genaev et al., 2019), as well as the general shape and spike trait characteristics. These traits characterise a complex view of the morphology (phenotype) of the spike by describing its shape (Circularity, Roundness), the physical dimensions of the ear body (Perimeter, Rachis length) and the area of the awns (Awns area), the traits obtained by approximating the ear by two quadrangles are related to the width (xu2, ybm) and length (xb2, yu2) of individual segments of the ear (Table 2).

**Table 2.**
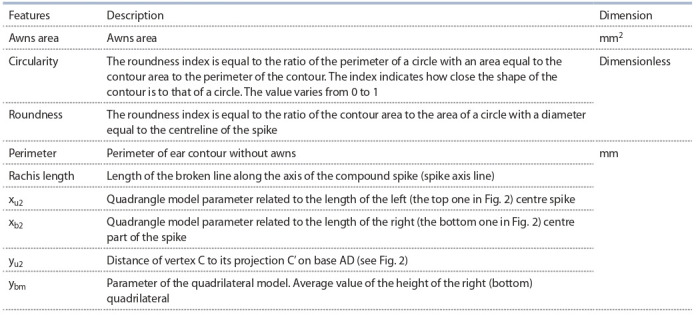
Description of the spike trait characters

**Data analysis.** In order to estimate the distribution of spikes in the feature space under study, we used a non-linear t-SNE dimensionality reduction algorithm (t-distributed stochastic neighbor embedding; Maaten, Hinton, 2008). This method allows to visualize multidimensional data by mapping objects in multidimensional space to a smaller (two- or three-dimensional) space. The basic idea behind t-SNE is to reduce the dimensionality of space while maintaining the relative pairwise distances between objects. The advantage of the t-SNE method is its tendency to localize isolated, dense spatial structures of arbitrary geometry. The t-SNE method was applied to ordinate images of spikes; the images of each of the projections of a single ear were treated as separate objects.

In order to assess the similarity of the quantitative characteristics of spikes for different species, we used hierarchical clustering (Johnson, 1967) of 17 wheat species according to the traits obtained by averaging over all spikes of the same species. Each species was characterized by a feature vector of length 9. A value of 1– r was used as a metric for the distance between species, where r is the value of the Pearson correlation coefficient between the values of the traits (Müllner, 2011). The linkage (UGMA algorithm) and dendrogram functions from the SciPy library (Virtanen et al., 2020) were used for clustering and dendrogram construction.

To compare the variance of traits in plants belonging to different ploidy types, we used F statistics (Snedecor, Cochran, 1989), which evaluates the significance of differences in the variance of two distributions. The data were normalized by the StandardScaler function of the scikit-learn library (Pedregosa et al., 2011). The test was performed independently for each of the nine traits described in Table 2. In this test, one spike image per plant was used, obtained in the “on the table” projection protocol.

## Results and discussions

The mean, median, standard deviation and variance of the nine features calculated for the 17 wheat species are presented in the Supplementary 1^1^.

^1^ Supplementary materials 1–4 are available in the online version of the paper: http://vavilov.elpub.ru/jour/manager/files/SupplPronozinA_Engl.pdf


Let’s review the distribution of spikes in our sample of plants according to the characteristic “area of the spikes”. The higher this parameter, the more awns were identified for the spike in the image. According to this characteristic, spikes of hexaploid wheat can be conditionally divided into three classes: awned (parameter value above 90), moderately awned (parameter value from 30 to 90), and awnless (parameter value below 30). The species T. compactum, T. spelta, T. petropavlovskyi and T. vavilovii are considered awned according to this criterion. T. aestivum, T. yunnanense, T. macha are moderately awned. The awnless ones are T. antiquorum, i:ANK-23, T. spelta cv. Rother Sommer Kolben, and T. sphaerococcum (see Supplementary 1). These data agree well with the appearance of the spikes (see Fig. 1, a). Thus, representatives of hexaploid wheat show considerable diversity in the presence/absence of awns.

If the classification above is applied to tetraploid wheat, only representatives of T. militinae (mean value of the parameter 24.09 mm2) can be assigned to the awnless category. Four species can be classified as moderately awned: T. dicoccoides, T. polonicum, T. timopheevii and T. turgidum. Three species are considered awned: T. aethiopicum, T. dicoccum, T. durum. In general, the representation of awned species (specimens) in tetraploid species is significantly higher than in hexaploid species.

Analysis of such characteristic as spike length shows that spikes can also be divided into three classes: length less than 60 mm (short), from 60 to 90 mm (medium) and more than 90 mm (long). According to this classification, the hexaploid wheat species T. spelta, T. petropavlovskyi and T. vavilovii can be classified as long spikes, T. aestivum, T. yunnanense, T. spelta cv. Rother Sommer Kolben and T. macha to medium spikes, and T. compactum, T. antiquorum, T. sphaerococcum and the near-isogenic lineage ANK-23 to short spikes. The boundary between species characterized by long and medium spikes is rather conditional. For tetraploid species we did not find any species which according to this parameter would fall into the category of long-boned. The mediumsized category could include T. aethiopicum, T. dicoccoides, T. polonicum, T. turgidum, the short spike category – T. dicoccum, T. durum, T. timopheevii and T. militinae.

**Fig. 3.**
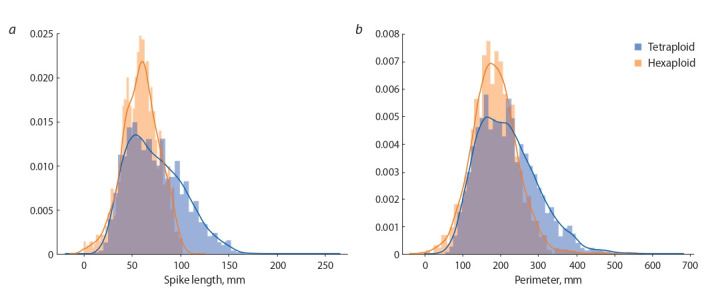
Length (а) and perimeter (b) distribution of a wheat spike in tetraploid (blue) and hexaploid (orange) wheat species.

The spike length distribution of the samples studied for hexaploid and tetraploid species is shown in Fig. 3, a. The Fig. 3, b shows the distribution of the parameter also characterizing the size of the spikes – the perimeter of the contour of the body of the spike in the image.

Fig. 3 shows that the distributions of both parameters in hexaploid wheat are more scattered, while the variability of these traits in hexaploid wheat is higher mainly due to the higher frequency of occurrence of ears with high values of these traits. 

**Fig. 4.**
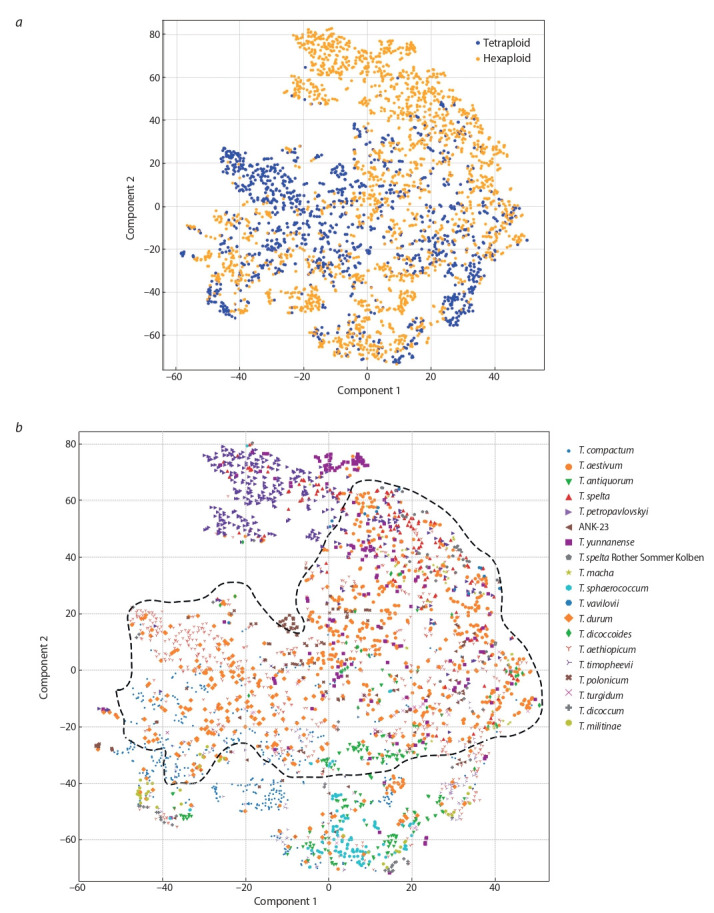
Clustering of spike digital images of individual genotypes by the t-SNE method, obtained on the basis of quantitative traits from Table 2. а – blue color corresponds to tetraploid wheat species, orange – hexaploid; b – the color and shape of each point corresponds to a specific type. The blue polygon marks the area occupied by T. aestivum and T. durum species. Clustering is called automatic partitioning into clusters. The automatic arrangement on the plane and in space is called ordination.

The distribution of the analyzed ears images in the space of nine features was visualized using the t-SNE method, resulting in a two-dimensional parameter space (components 1 and 2). The results of the transformation are shown in Fig. 4. In the resulting diagram, each point represents one of the analysed images of the spike. In Fig. 4, a the dots are coloured according to the type of ploidy of the plant (blue colour corresponds to tetraploid wheat species, orange to hexaploid ones). In Fig. 4, b the colour and shape of each dot corresponds to a particular wheat spike image.

The diagram in Fig. 4, a shows that the areas occupied by hexa- and tetraploid wheat species strongly overlap on the graph. This means that the spikes of these two groups are quite similar in their characteristics. This is consistent with the results presented in the Supplementaries 1 and 2 as well as in Fig. 3. However, it should be noted that in the diagram in Fig. 4, a samples of hexaploid species occupy a larger area, primarily due to the predominance of the corresponding points in the right part of the diagram. One can see that orange dots (hexaploid wheat) predominate in the area with values of component 1 more than –20, this predominance is even more pronounced in the upper right corner of the diagram (values of component 1 less than 0 and component 2 more than 20). This means that a number of spike trait characteristics have some values for hexaploid species specific only, but not for tetraploid ones. This agrees well with the result shown in Fig. 3. In particular, such areas may correspond to large values of the parameters “perimeter” and “ear length”.

The diagram in Fig. 4, b shows that the areas occupied by samples of the different species overlap considerably. For example, T. aestivum and T. durum species overlap across the entire plot area (dotted line). At the same time, it should be noted that the images of spikes belonging to the same wheat species occupy mostly compact areas on the graph. At the same time, there are species for which the spike samples are divided into several clearly visible clusters according to their characteristics. Such species include T. compactum (small blue circle, component 1 from –60 to 0, component 2 from –60 to 0) and T. petropavlovskyi (purple triangle, component 1 from –20 to 0, component 2 from 40 to 80).

Fig. 1 shows that hexaploids are represented by plants with two characteristic types of spikes: long and thin (T. aestivum, T. spelta, T. petropavlovskyi, T. yunnanense, T. spelta cv. Rother Sommer Kolben); short and rounded (T. compactum, T. antiquorum, i:ANK-23, T. sphaerococcum, T. macha, T. vavilovii). In Fig. 4. b the group of plants with short and rounded spikes is located in the component 2 value range from –80 to 0 (lower part of the graph). Plants with long and thin spikes have component 2 values between 0 and 80 (upper part of graph). In Fig. 4, a, these two groups of plants correspond roughly to the two clouds of dots in hexaploid wheat at the top and bottom of the graph, which overlap slightly in the central part of the graph. Thus, the diagrams in Fig. 4 provide a clear indication of the diversity of spikes in their characteristics within and between species.

**Fig. 5.**
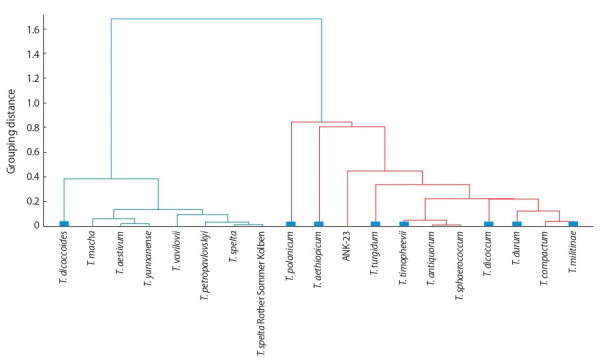
Results of the hierarchical cluster analysis for nine signs of a wheat spike. Blue squares correspond to tetraploid.

To characterize in more detail the similarity of morphometric characteristics of spikes in different wheat species, we conducted a hierarchical cluster analysis for them based on a comparison of the mean values of the studied traits. (Fig. 5)

Fig. 5 shows that the wheat species were divided into two clusters (highlighted in red and green). The first cluster (red) predominantly includes tetraploid species (shown in blue rectangles near the terminal tree nodes). However, wild tetraploid wheat species T. diccocoides is not included in this cluster, while among hexaploid species, T. compactum, T. antiquorum and T. sphaerococcum differing from all other species by compact spike shape, i. e. having the shortest spike of all studied hexaploid wheat species are included in it. It should be noted that in the work of A. Zatybekov et al. (2020), using economically important traits, samples of six tetraploid species were clustered arbitrarily, i. e. irrespective of their species identity. It is important to note, that remaining hexaploid species were clearly divided by spike length into two clusters of medium (T. macha, T. aestivum, and T. yunnanense) and long spikes (T. vavilovii, T. petropavlovskyi, and T. spelta).

T. spelta and T. spelta cv. Rother Sommer Kolben (a German landrace) occur in the same cluster. This allows us to conclude that the “species” shape of spike during long-term wheat breeding did not change for a long time (in this case, more than fifty years) and may be successfully used for classification of the species.

It should be noted that the only wild tetraploid loose spike species in the genus, T. dicoccoides, has fallen to the hexaploids. While hexaploid wheat species with compact ear type – T. compactum, T. antiquorum, T. sphaerococcum and human-made near-isogenic line ANK-23 of spring bread wheat cv. Novosibirskaya 67 (Koval, 1997) – were included into tetraploid species. The latter leads to the conclusion that although near-isogenic lines are produced on a particular (specific) species, nevertheless, their species identity should be treated with caution.

Let’s take a look at T. petropavlovskyi. The species was founded at the Chinese Pamir – route of the Great silk road. According to the results of the study of gliadins, all accessions of this species were very similar to such hybrid combination obtained from crossing bread wheat with T. polonicum (Watanabe et al., 2004). The authors of the “Cultural Flora of the USSR” also considered a possible hybrid origin of this species (Dorofeev et al., 1979). In addition, T. petropavlovskyi also resembles bread wheat in a number of taxonomic traits (Goncharov, 2005). Previously, R.L. Boguslavsky (1982) described hybrids from crossing T. aestivum with T. polonicum produced by CIMMYT breeders as subspecies of T. petropavlovskyi ssp. mexicana Bogusl. Based on the above, we considered it appropriate to conbided T. petropavlovskyi as the subspecies of T. aestivum:

- **Triticum aestivum ssp. petropavlovskyi comb. et stat. nov. (Udacz. et Migusch.) N.P. Gontsch.** – T. turanicum Jakubz. convar. montanostepposum Jakubz. f. aristiforme Jakubz. 1959. Bot. Zhur. 10:1428, nom. illig. – T. petropavlovskyi Udacz. et Migusch. 1970. Vestn. Sel’skokhoz. Nauki. 9:20.
- **Typus:** described by an accession from China “Origin: China, Xinjiang Province, village Kurlia, K-48376, 1957. A.M. Gorsky exp[edition]. Reproduction of Central Asia, Tashkent, Central Asian Station of VIR. 08. VII. 1969, Collected/defined: R.A. Udachin & E.F. Migushova” in St. Petersburg (WIR!). (The herbarium specimens of the type and paratype of Triticum aestivum ssp. petropavlovskyi are given in the Supplementaries 3 and 4).

Note that the results presented in Fig. 3 and 4, a show that hexaploid species have a greater variability in spike shape, size and awnness characteristics. Therefore, we hypothesized that the spike trait characteristics of hexaploid species may have a higher variation than those of tetraploid species. To test this assumption, we compared the variance of the estimated parameters using an F-distribution (Table 3).

**Table 3.**
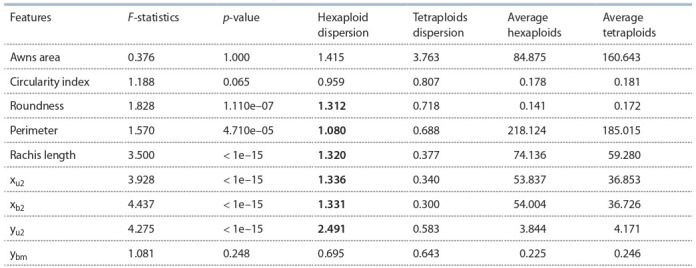
Results of using F statistics to confirm the hypothesis of a significant difference in the variance of two distributions Notе. Significant dispersion differences are shown in bold

The results presented in Table 3 show that the variance of most of the characters for hexaploids and tetraploids have significant differences ( p < 0.05). At the same time, the significant differences in variance were not found for such traits as ybm (quadrangle model parameter), Awns area and Circularity index. It is interesting to note that for all significant differences, we observe a higher variance in hexaploids than in tetraploids. Thus, the analysis showed that hexaploid species show higher diversity in spike morphometric trait characteristics compared to tetraploid species.

The data represent plants of 17 wheat species: 9 hexaploids (T. compactum, T. aestivum, T. antiquorum, T. spelta (including T. spelta cv. Rother Sommer Kolben), T. petropavlovskyi, i:ANK-23 (near-isogenic line of bread wheat cv. Novosibirskaya 67), T. yunnanense, T. macha, T. sphaerococcum, T. vavilovii) and 8 tetraploids (T. aethiopicum, T. dicoccoides, T. dicoccum, T. durum, T. militinae, T. polonicum, T. timopheevii, T. turgidum). The results of their clustering are presented so that the colour and shape of each dot corresponds to a particular species (see Fig. 5).

It is well known that genome doubling as a result of duplications (autopoploidy) or hybridization and subsequent polyploidization (alloploidy) leads to marked changes in plant phenotype (Finigan et al., 2012; Romanov, Pimonov, 2018; Rodionov et al., 2019). These changes in plants occur both at the cellular level (Liu et al., 2018) and at the organ level (Robinson et al., 2018). In many cases, in plants, an increase in ploidy leads to an increase in cell and organ size (Comai, 2005; Williams, Oliveira, 2020), increasing resistance to stress (Tan et al., 2015). Currently, researchers suggest that there are four types of molecular mechanisms of such variability: 1) increased gene/allele dosage, 2) increased genetic diversity, 3) altered genetic regulation, and 4) epigenetic rearrangements of the genome (Chen, 2007; Finigan et al., 2012). 

The analysis of morphological characteristics of spikes of hexaploid (2n = 6x = 42) and tetraploid (2n = 4x = 28) wheat has shown, that most of spike characteristics have significantly higher variation in wheat with higher spike ploidy. Our results are in agreement with the ideas about the influence of ploidy on plant phenotype variability.

## Conclusion

A large-scale analysis of the spike digital images of 595 plants of 8 tetra- and 9 hexaploid wheat species was carried out. Nine quantitative traits describing the shape, size and awnedness of the spike were studied. The variability among the above genotypes was studied and it was shown that two clusters are formed in the spike characteristic space. The first cluster includes mainly hexaploid species (with the exception of wild tetraploid species T. dicoccoides). The second cluster includes tetraploid species (with the exception of three hexaploid species with compact spike shape – T. antiquorum, T. sphaerococcum, and near-isogenic line ANK-23). Analysis of variance of these characters in hexaploid and tetraploid wheats showed a significant in crease in variance for six of nine characters in the sample of hexaploids, i. e. greater ploidy level gives more variability in quantitative characters of spike morphology

Thus, it is shown that morphological trait characteristics of spikes of hexa- and tetraploid species, obtained on the basis of computer image analysis, demonstrate the differences, which can be used in the future to develop a method of classification of plants by ploidy level and their species affiliation in automatic mode.

## Conflict of interest

The authors declare no conflict of interest.
